# The Use of Plaster of Paris in Fractures

**Published:** 1882-03

**Authors:** 


### THE USE OF PLASTER-OF-PARIS IN FRACTURES.

Plaster, either in the form of a bandage enveloping the fractured part, or in the form of a distinct splint, is used quite extensively in the various hospitals of this city. In fact, all other things being equal, it is given the preference over other forms of apparatus usually employed in such injuries. Particularly is this the case with fractures of the leg, which are treated now almost exclusively by this bandage. The fracture-box is rarely used, and only in exceptional cases, where there is great swelling, and under conditions of extensive injury of the skin, in which it is necessary for the parts to be exposed during treatment. Generally this open method is only employed until such time as it is safe to apply the plaster-of-Paris bandage, as shown by the disappearance of the swelling and the healing of the abrasions. No time is lost in so doing, as generally the parts are made fit for the immovable apparatus before the bony union commences. In compound fracture the limb is generally placed at once in the plaster apparatus, openings being made in the latter corresponding with the injuries of the soft parts, for the purpose of establishing thorough drainage. As a rule, and when, of course, there is no special contra-indication in the shape of undue swelling, etc., all fractures in which plaster-of-Paris is to be employed are "put up" at once. A general description of the method of procedure may apply to that to be employed in any case of fracture in any region of the body. The part is enveloped in a thin layer of cotton and the bandages, immersed in water sufficiently long to be permeated, are applied directly over the cotton, care being taken to exert slight and uniform pressure. Each layer of bandage is carefully moulded to the inequalities of the surface, and made perfectly smooth before the next layer is applied. If the bandages are properly prepared, without sizing, and have been kept in a dry place, the plaster will commence to "set" before the second bandage is applied. Generally three layers of bandage are sufficient for a fracture where ordinary support is required. Four, with suitable reinforcements, may be required in other cases. After the dressing is complete, it is exposed to the air, and hardens sufficiently in two or three hours to allow the limb to be moved.

The plaster apparatus is generally kept in position during the whole period of treatment. If undue swelling occurs, the envelope is slit in the long axis of the limb by a Hays saw, or by a scissors for the purpose, and thus a splint is formed which is kept in position by outside bandages.

Some surgeons prefer to dispense with cotton altogether, and use a well fitted silk or gauze stocking or jacket as the foundation for the plaster. There is, however, greater care and skill required in this method, as any undue pressure at any one point would be more apt to produce swelling in the parts beyond. Yet still, when properly applied, this makes the most comfortable and lightest dressing that can be used, and gives the perfection of support and greatest accuracy of adjustment to the injured parts.—*Medical Record.*

